# Etiological Detection, Isolation, and Pathogenicity of Porcine Reproductive and Respiratory Syndrome Virus in China

**DOI:** 10.3390/vetsci12060530

**Published:** 2025-05-29

**Authors:** Yingbin Du, Jingyi Chen, Tianze Ren, Chunying Xie, Yiye Zhang, Liurong Fang, Yanrong Zhou

**Affiliations:** 1 National Key Laboratory of Agricultural Microbiology, College of Veterinary Medicine, Huazhong Agricultural University, Wuhan 430070, China; 2 Key Laboratory of Preventive Veterinary Medicine in Hubei Province, the Cooperative Innovation Center for Sustainable Pig Production, Wuhan 430070, China

**Keywords:** porcine reproductive and respiratory syndrome virus, NADC30/34-like strain, genetic variation, phylogenetic analysis, pathogenicity

## Abstract

Due to its high genomic variability, the epidemiological landscape of porcine reproductive and respiratory syndrome virus (PRRSV) has become increasingly complex in recent years. From 2022 to 2023, we collected a total of 1044 clinical samples from pigs suspected of PRRSV infection in China and discovered a PRRSV-positive rate of 29.8% (311/1044) using RT-PCR targeting the nsp2 gene. Among these positive samples, NADC30/34-like PRRSV, highly pathogenic PRRSV (HP-PRRSV), and classical PRRSV strains accounted for 60.1%, 37.9%, and 4.5%, respectively. These results indicate that the most prevalent PRRSV strains in China are NADC30/34-like PRRSV, followed by HP-PRRSV. Two PRRSV strains, JX03 and HN08, were isolated, and TCID_50_ assays were performed to determine their titers at different time points post-infection, revealing differences in their proliferation kinetics. Phylogenetic, amino acid sequence, and recombination analyses demonstrated that the JX03 and HN08 strains cluster within lineage 8 (HP-PRRSV) and sublineage 1.5 (NADC34-like PRRSV), respectively. Notably, the HN08 strain was identified as a recombinant between the NADC30-like and NADC34-like strains, while no recombination event was detected in the JX03 strain. Pathogenicity assessments showed that the JX03 strain exhibited higher pathogenicity than the CHN-HB-2018 strain (a NADC30-like PRRSV strain was previously isolated by our lab), as evidenced by differences in clinical signs and mortality rates in piglets. In contrast, HN08 displayed no obvious clinical symptoms or mortality, revealing lower pathogenicity than the CHN-HB-2018 strain. These findings provide valuable information on the epidemiological and genetic characteristics of PRRSV strains in China, laying a foundation for the development of effective strategies against PRRSV.

## Simple Summary

Porcine reproductive and respiratory syndrome (PRRS) is a global widespread infectious disease that severely impacts the swine industry. The high genetic variability of the PRRS virus (PRRSV) has led to the continuous emergence and spread of novel strains. The surveillance of PRRSV epidemiology and isolation of circulating strains are crucial for developing effective PRRSV control strategies. In this study, we confirmed that NADC30/34-like PRRSV is the dominant epidemic strain in China, followed by highly pathogenic PRRSV (HP-PRRSV). An HP-PRRSV strain (JX03) and a recombinant NADC34-like PRRSV strain (HN08) were isolated, and their whole genome and phylogenetic characterization were analyzed. Animal experiments confirmed that the JX03 strain exhibited the highest pathogenicity, followed by CHN-HB-2018 (a NADC30-like PRRSV strain previously isolated by our lab), while HN08 demonstrated the lowest pathogenicity. These findings provide critical information for PRRSV molecular epidemiology and vaccine strain selection.

## 1. Introduction

Porcine reproductive and respiratory syndrome (PRRS) is a highly contagious viral disease that causes significant economic losses in the global swine industry [1,2]. The causative agent, PRRSV, is a single-stranded positive-sense RNA virus belonging to the Arteriviridae family within the order Nidovirales [3]. The PRRSV genome is approximately 15 kb in length and encodes at least 10 open reading frames (ORFs), including ORF1a, ORF1b, ORF2a, ORF2b, ORF3, ORF4, ORF5, ORF5a, ORF6, and ORF7 [4,5]. Notably, ORF1a and ORF1b encode the polyproteins pp1a and pp1ab, which are subsequently cleaved by proteases into at least 16 nonstructural proteins (NSPs) [6]. ORFs 2–7 encode structural proteins, including GP2, envelope protein (E), GP3-GP5, GP5a, membrane protein (M), and nucleocapsid protein (N) [2].

The PRRSV genome exhibits high genetic variability. In 2016, the International Committee on the Taxonomy of Viruses (ICTV) formally classified PRRSV as two distinct viral species, PRRSV-1 and PRRSV-2 [7]. Based on the ORF5 gene sequences, PRRSV-1 is further categorized into four lineages, while PRRSV-2 is classified into eleven lineages [8,9]. In China, PRRSV-2 is the predominant circulating strain, with PRRSV-1 being relatively uncommon [10,11,12,13]. Therefore, most epidemiological studies on PRRSV in China primarily focus on PRRSV-2. Currently, PRRSV-2 in China is primarily concentrated in lineage 1 (NADC30/34-like PRRSV), lineage 3 (QYYZ-like PRRSV), lineage 5 (VR-2332-like PRRSV), and lineage 8 (HP-PRRSV), with lineages 1 and 8 being the most prevalent [10,14,15,16,17]. Detection rates of NADC30-like and NADC34-like strains have recently increased dramatically, exhibiting widespread distribution across multiple Chinese provinces [18,19,20,21].

Various PRRSV strains exhibit distinct pathogenic characteristics [10,22,23,24]. HP-PRRSV is characterized by severe clinical manifestations, such as high fever and elevated mortality rates in piglets [25]. NADC30-like isolates exhibit marked pathogenicity divergence [13,22,26,27,28]. Most NADC30 strains caused transient fever without mortality in infected pigs [29], while the NADC30-like PRRSV JL580 strain demonstrated high virulence, causing severe clinical manifestations including persistent pyrexia, respiratory distress, and 100% mortality [28]. Similar pathogenic heterogeneity occurs in NADC34-like strains. Comparative studies have revealed that the majority of NADC34-like strains, such as IA/NADC34, IA/ISU-1, and IA/ISU-5, induced severe interstitial pneumonia with pulmonary edema and hemorrhagic lesions, whereas IA/ISU-2 and HLJDZD32-1901 caused only mild pulmonary pathology or limited interstitial pneumonia [30,31]. Diverse recombination patterns serve as a key determinant of pathogenicity differences among NADC30/34-like variants. For example, the recombinant strain SDLY23-1742 resulting from NADC30-like, NADC34-like, and HP-PRRSV genetic recombination exhibited higher pathogenicity than BJ1805-2 (a NADC34-like strain with no recombination event) [24]. Consequently, continuous monitoring of PRRSV-2 prevalence and clinical pathogenic characteristics remains essential for developing effective prevention and control strategies.

The nsp2 gene is frequently utilized in molecular epidemiological investigations due to its characteristic amino acid deletions and insertions [15,18,20,32]. Compared with the original strain of PRRSV-2 (VR-2332), HP-PRRSV exhibits a discontinuous 30-aa (1 + 29) deletion, while NADC30-like PRRSV displays a more extensive discontinuous deletion of 131-aa (111 + 1 + 19), and the emerging NADC34-like strains feature a contiguous 100-aa deletion [15,33,34]. Similarly, the PRRSV ORF5 gene also exhibits high genetic diversity [16,35]. Classifying PRRSV into lineages or clades through phylogenetic analysis of the ORF5 gene is essential for understanding the genetic relationships among different PRRSV strains [30,36,37]. In the clinical surveillance of PRRSV, simultaneous targeting of both the nsp2 and ORF5 genes for genetic variation analysis significantly enhances detection accuracy and provides comprehensive molecular epidemiological characterization [38].

In this study, we conducted an epidemiological analysis of PRRSV in China from 2022 to 2023. Our findings revealed that the currently prevalent strains are predominantly NADC30/34-like PRRSV, followed by HP-PRRSV. An HP-PRRSV strain (JX03) and a recombinant NADC34-like PRRSV strain (HN08) were isolated. Phylogenetic, amino acid sequence, and recombination analyses demonstrated that the JX03 and HN08 strains cluster within lineages 8 and 1.5, respectively. A comparison of pathogenicity demonstrated variations among different PRRSV lineages, with JX03 exhibiting the highest pathogenicity, followed by CHN-HB-2018, while HN08 had the lowest pathogenicity. These findings provide insights into the molecular characteristics, genetic diversity, pathogenicity, and evolution of PRRSV in China.

## 2. Materials and Methods

### 2.1. Clinical Sample Collection and Treatment

Clinical tissue samples, including lungs, placentas, and aborted fetuses, were collected from pigs suspected of being infected with PRRSV across 27 provinces (including municipalities and autonomous regions) in China. These samples (0.5 g) were homogenized in 2 mL of phosphate-buffered saline (PBS) using a tissue homogenizer for 10 min, followed by centrifugation at 12,000 rpm for 10 min at 4 °C. The supernatants were aliquoted and stored at −80 °C for RNA extraction and virus isolation.

### 2.2. RNA Extraction and RT-PCR Detection

Total RNA was extracted and reverse transcribed into cDNA via the TRIzol (Omega, Georgia, USA) method and an RNA reverse transcription kit (Vazyme, Nanjing, China) following the manufacturer’s instructions. PCR was subsequently performed using a pair of specific primers (forward: 5′-CTCCTTTGATTGGAATGTTGTG-3′; reverse: 5′-GATGGCTTGAGCTGAGTATTTTG-3′) to amplify a gene fragment within the nsp2 region, which allows for the differential detection of NADC30/34-like PRRSV (681 bp), HP-PRRSV (984 bp), and classical PRRSV (1072 bp) strains [39].

### 2.3. Virus Isolation and Growth Curve Detection

Porcine alveolar macrophages (PAMs) were cultured in Roswell Park Memorial Institute 1640 medium (RPMI-1640; Sigma, St. Louis, MO, USA) supplemented with 10% fetal bovine serum (FBS). The prepared supernatants of PRRSV-positive samples were filtered through a 0.22 μm filter (Millipore, Burlington, MA, USA). Subsequently, PAMs seeded in 12-well plates were inoculated with the resulting filtrates and incubated for 2 h at 37 °C. Following inoculation, the cells were washed three times with PBS to eliminate the remaining inoculum, supplemented with fresh medium, and incubated for an additional 2 to 3 days prior to harvest. Next, the harvested cell cultures underwent three freeze–thaw cycles to facilitate viral release. The samples were then centrifuged at 12,000 rpm for 10 min, and the supernatant was collected as the F0 generation virus. Three serial passages were conducted following the same procedure. Finally, the isolated viruses were stored at −80 °C.

For viral growth curve detection, PAMs in 12-well plates were infected with PRRSV isolates at a 0.01 multiplicity of infection (MOI) for 2 h (37 °C). After removing the inoculum and three washes with PBS, the plates were supplemented with fresh medium. At 6, 12, 24, 36, 48, 60, and 72 h post-infection (hpi), the supernatant and cells were harvested, and 50% tissue culture infectious dose (TCID_50_) assays were performed to determine viral titers.

### 2.4. Indirect Immunofluorescence Assay (IFA)

PAMs infected or mock-infected with PRRSV isolates were washed with PBS, fixed with 4% paraformaldehyde, and permeabilized with cold methanol. After being blocked with 5% bovine serum albumin, the cells were incubated with a PRRSV-specific monoclonal antibody (mAb) against N protein (previously generated in our lab), followed by a fluorescein isothiocyanate (FITC)-conjugated goat anti-mouse IgG antibody (Invitrogen, Carlsbad, CA, USA). Finally, the nuclei were stained with 0.01% 4′,6-diamidino-2-phenylindole (DAPI), and the fluorescence images were visualized using a fluorescence microscope (Olympus, Tokyo, Japan).

### 2.5. Multiple Alignment, Phylogenetic, and Recombination Analyses

The complete genome sequences of the isolated strains were determined with the paired primers listed in Table S1. Specific primers (forward: 5′-ATGTTGGGGAAATGCTTGACC-3′; reverse: 5′-CTAGAGACGACCCCATTGTTC-3′) targeting the ORF5 gene were used for RT-PCR amplification, followed by sequencing. A total of 51 complete genome sequences and 70 ORF5 gene sequences of PRRSV were retrieved from the NCBI GenBank database https://www.ncbi.nlm.nih.gov/ (accessed on 4 September 2024) to serve as reference sequences. Detailed information about these sequences is provided in Table S2. Phylogenetic analyses were constructed for both whole-genome sequences and ORF5 gene sequences. Sequence alignment was conducted using MAFFT v7.526 [40]. For homology analysis, the MegAlign program within DNASTAR v7.0 was employed [41]. Maximum likelihood (ML) phylogenetic trees were generated using MEGA v12.0. Additionally, the amino acid sequences of the nsp2 and GP5 proteins were analyzed using CLC Main Workbench v6.8. Recombination analyses were performed for the nucleotide sequence of PRRSV isolates using RDP v4.0.

### 2.6. Animal Experiments

Twenty-four 4-week-old piglets were confirmed negative for both PRRSV antigen and antibodies and randomly assigned to four groups, with six piglets in each group. The first group was infected with the JX03 strain (2 × 10^5^ TCID_50_ per piglet), while the second and third groups were infected with the HN08 and CHN-HB-2018 strains, respectively (2 × 10^6.5^ TCID_50_ per piglet for both groups). The fourth group (negative control) was administered an equivalent volume of RPMI-1640. All groups were inoculated intramuscularly (1 mL) and intranasally (1 mL), respectively.

Following viral infection, the piglets were monitored daily for rectal temperature, and body weight was recorded once weekly. Serum and nasal swabs were collected at 0, 3, 5, 7, 10, and 14 days post-infection (dpi). At 5 and 10 dpi, one piglet from each group was randomly euthanized for necropsy. RT-qPCR was used to determine viral loads in serum, nasal swabs, and tissues, as described previously [42]. At necropsy, lung and hilar lymph node samples were collected and subsequently fixed in formalin for histopathological analysis and immunohistochemistry (IHC). IHC was performed with a PRRSV N-specific monoclonal antibody.

### 2.7. Statistical Analyses

Statistical analyses were performed using GraphPad Prism v10.1.0. Multiple comparisons were conducted using a one-way analysis of variance; data are presented as means ± standard deviations.

## 3. Results

### 3.1. RT-PCR Detection and Phylogenetic Analysis of PRRSV in China

**Figure 1:**
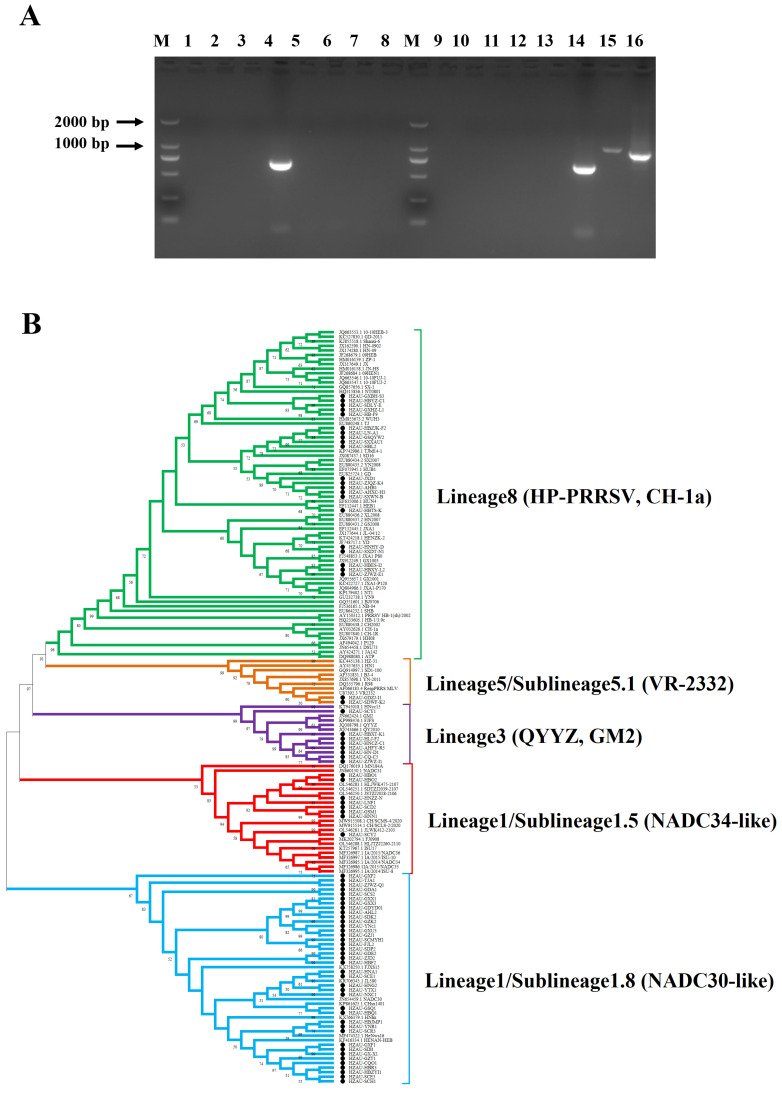
PRRSV detection of clinical samples. (**A**) RT-PCR detection (partial) results for PRRSV in China (2022.6–2023.6). M: 2000 bp DNA marker; lane 1–16: clinical samples of PRRSV; lane 4, 14: NADC30/34-like PRRSV (681 bp); lane 15: classical PRRSV (1072 bp); lane16: HP-PRRSV (984 bp). (**B**) Genetic evolution analysis of PRRSV-positive samples based on nucleotide sequences of ORF5 gene. Phylogenetic trees consist of five subgroups, including lineage 1/sublineage 1.5 (blue), lineage 1/sublineage 1.8 (red), lineage 3 (purple), lineage 5/sublineage 5.1 (orange), and lineage 8 (green). Positive clinical samples are highlighted by black circles.

From June 2022 to June 2023, a total of 1044 clinical tissue samples, including lung, placenta, and aborted fetuses, were collected from pigs suspected of infection with PRRSV across 27 provinces (including municipalities and autonomous regions) in China. The samples were tested by RT-PCR to amplify a gene fragment within the nsp2 region, allowing for the differential detection of NADC30/34-like PRRSV, HP-PRRSV, and classical PRRSV strains (Figure 1A). As shown in Table 1, the overall positive rate for PRRSV was 29.8% (311/1044). Among the positive samples, NADC30/34-like PRRSV, HP-PRRSV, and classical PRRSV accounted for 60.1% (187/311), 37.9% (118/311), and 4.5% (14/311), respectively. Moreover, co-infections involving NADC30/34-like and HP-PRRSV strains were observed, with an infection rate of 2.6% (8/311).

To further investigate the epidemiological trends of PRRSV strains, ORF5 genes were amplified and sequenced from positive samples, yielding 78 ORF5 gene sequences, which were then used for phylogenetic analysis. The results showed that these sequences clustered into four lineages, including lineage 1, lineage 3, lineage 5, and lineage 8, with proportions of 60.26% (47/78), 2.56% (2/78), 10.26% (8/78), and 26.92% (21/78), respectively (Figure 1B). RT-PCR results based on nsp2 and a phylogenetic analysis based on ORF5 confirmed that the predominant circulating PRRSV strains in China are primarily of lineage 1 (NADC30/34-like PRRSV), followed by lineage 8 (HP-PRRSV).

### 3.2. Isolation and Identification of PRRSV Epidemic Strains

**Figure 2:**
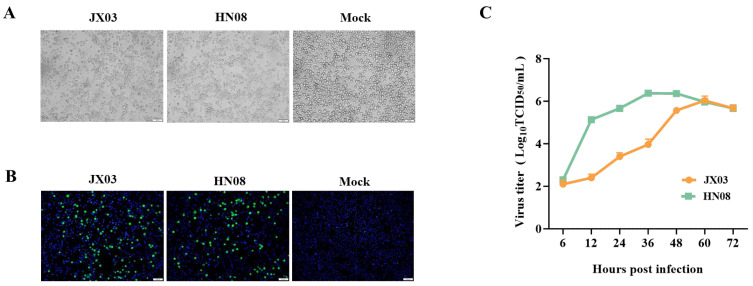
Isolation and identification of the PRRSV strains JX03 and HN08. (**A**) Cytopathic effects (CPEs) in PAMs infected with JX03 and HN08 strains. (**B**) Identification of JX03 and HN08 strains in PAMs by indirect immunofluorescence assay with PRRSV N-specific mAb. Fluorescence images were captured via fluorescence microscope (Olympus). (**C**) Growth curve of JX03 strain and HN08 strain in PAMs.

To isolate PRRSV epidemic strains, PAMs were inoculated with the supernatant of tissue homogenates obtained from PRRSV-positive samples. Following a 2 h incubation, the inoculum was removed and replaced with fresh RPMI-1640. Typical CPEs of PRRSV were observed in two samples after about 48 h of inoculation (Figure 2A). IFA was performed using PRRSV N-specific mAb to further confirm the presence of PRRSV. As shown in Figure 2B, green fluorescence signals were observed in virus-infected PAMs, and no green fluorescence signals were observed in the control group.

Additionally, we also detected the viral titers of these two strains at different time points post-infection using the TCID_50_ assay and plotted the growth curve. The results showed that these two PRRSV strains exhibited distinct replication kinetics, with peak viral titers occurring at 36 and 60 hpi, respectively, followed by a gradual decline (Figure 2C). These findings collectively confirmed the successful isolation of two PRRSV strains, JX03 and HN08, respectively.

### 3.3. Whole-Genome Characterization and Phylogenetic Analysis of PRRSV Isolates

Twelve pairs of primers were designed to amplify and sequence the whole genomes of the JX03 and HN08 strains, respectively. The sequencing results revealed that the full-length genomes of the JX03 and HN08 strains were 15,024 and 15,325 nucleotides (nt), respectively, excluding the poly(A) tail. Homology analyses of the JX03 and HN08 strains were conducted with seven PRRSV reference strains, including Ch-1a, CH-1R, JXA1, NADC30, NADC34, GM2, and QYYZ. The results are presented in Table 2 and Table 3. The JX03 strain exhibited the highest whole-genome nucleotide homology with the HP-PRRSV strain JXA1 at 99.0%. Furthermore, the coding regions (nsp1–12 and ORF2–7) of the JX03 strain showed the highest nucleotide and amino acid homology with the corresponding regions of the JXA1 strain, ranging from 97.6% to 99.8% and from 97.3% to 100.0%, respectively (Table 2). Phylogenetic analysis based on whole-genome nucleotide sequences showed that the JX03 strain clusters within lineage 8 (HP-PRRSV). These homology and phylogenetic analyses further confirm that the JX03 strain belongs to HP-PRRSV.

**Figure 3:**
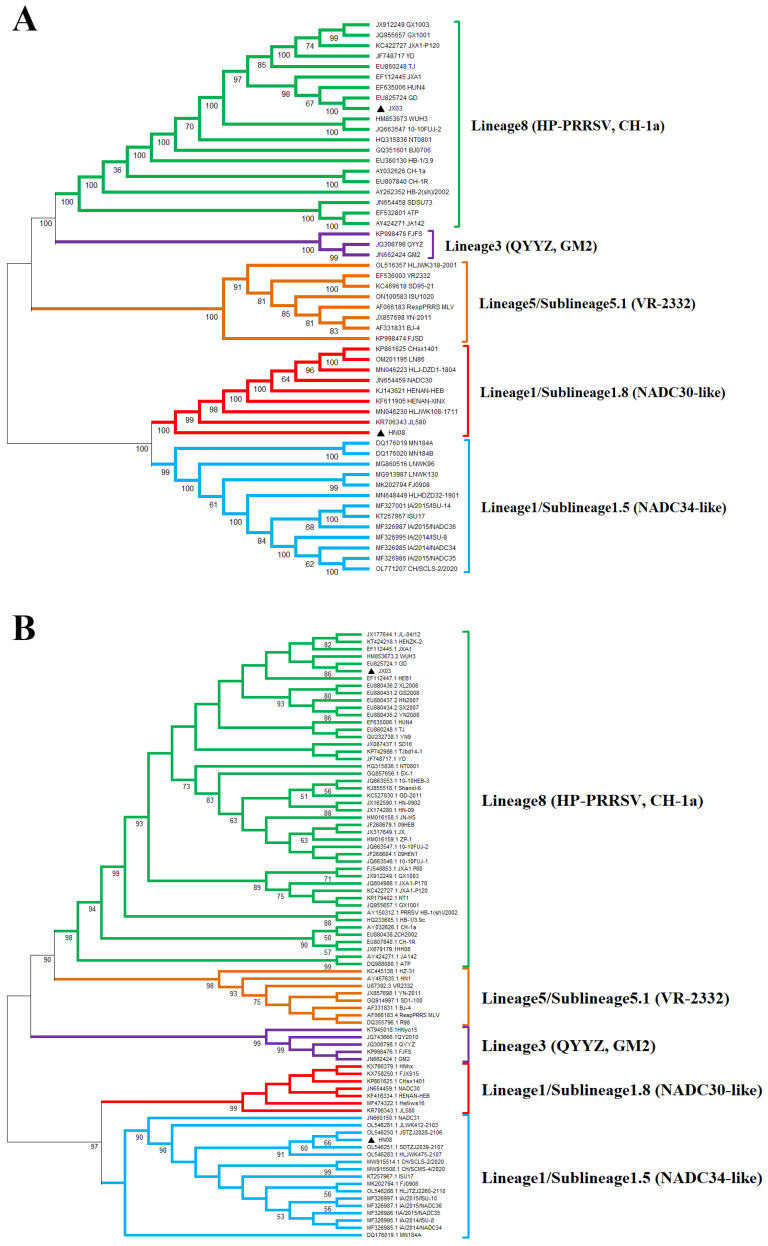
Genomic characterization analysis of JX03 and HN08 strains. Phylogenetic trees based on (**A**) complete genomes and (**B**) ORF5 nucleotide sequences of PRRSV. Phylogenetic trees consist of five subgroups, including lineage 1/sublineage 1.5 (blue), lineage 1/sublineage 1.8 (red), lineage 3 (purple), lineage 5/sublineage 5.1 (orange), and lineage 8 (green). Isolated strains are highlighted by black triangles.

Additionally, the HN08 strain had the highest whole-genome nucleotide homology with the NADC30 strain at 90.0%. Consistent with the homology analysis results, the phylogenetic tree constructed based on the whole-genome nucleotide sequences indicated that the HN08 strain belongs to sublineage 1.8 (NADC30-like PRRSV) (Figure 3A). Consistently, its ORF1a, ORF1b, and ORF6–7 regions also demonstrated the highest homology with the NADC30 strain. Nucleotide homology ranged from 81.5% to 94.1% and amino acid homology ranged from 80.6% to 97.7% (Table 3). Interestingly, the HN08 strain exhibited the highest homology with the NADC34 strain in the ORF2–5 regions. Nucleotide homology ranged from 92.9% to 95.9% and amino acid homology ranged from 92.2% to 97.0%, indicating that it belongs to the NADC34-like PRRSV. In line with nucleotide homology results, the phylogenetic tree constructed from the ORF5 gene nucleotide sequences revealed that the HN08 strain clusters within sublineage 1.5 (NADC34-like PRRSV) (Figure 3B). These results suggest that the HN08 strain may be a recombinant strain derived from NADC30-like PRRSV and NADC34-like PRRSV.

### 3.4. Gene Recombination Analysis of HN08

**Figure 4:**
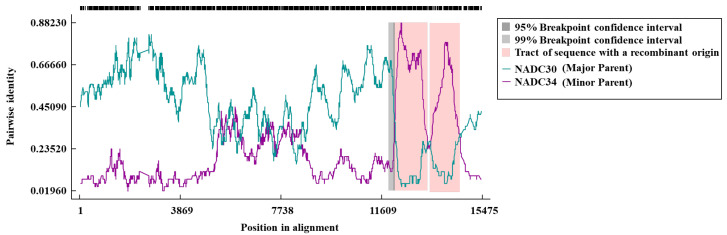
The genome recombination analysis of PRRSV HN08 strain. The Y-axis indicates the percentage similarity of the query sequence (HN08) to the NADC30 (blue) and NADC34 (purple) strains. The recombination area is highlighted in pink.

Phylogenetic and homology analyses indicated that the HN08 strain may be a recombinant strain. Therefore, a comprehensive recombination analysis of the HN08 strain was conducted using RDP 4.0 NADC30 PRRSV (GenBank: MH500776) and NADC34 PRRSV (GenBank: MF326985) as reference strains. The results revealed that the HN08 strain is a recombinant virus primarily derived from the NADC30 strain, with the NADC34 strain serving as a secondary parent. The recombinant regions were identified between nt 11,712–12,781 (ORF2–3 region) and nt 13,390–14,030 (ORF5 region). These results are consistent with the homology and phylogenetic analysis findings for the HN08 strain (Figure 4).

### 3.5. Amino Acid Variations in nsp2 and GP5 of PRRSV Isolates

**Figure 5:**
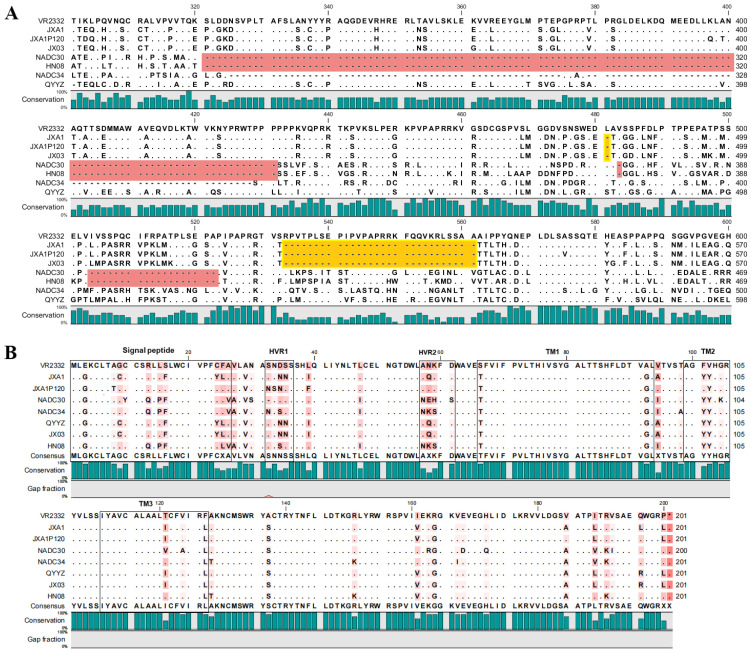
Amino acid sequence analyses of JX03 and HN08 strains. (**A**) Amino acid sequence analysis of nsp2 (300–600 aa) in JX03 and HN08 strains. Different colors denote the defined deletion pattern as follows: the “111 + 1 + 19” aa deletion pattern of NADC30-like PRRSV (red); the “1 + 29” aa deletion pattern of HP-PRRSV (yellow). (**B**) An amino acid sequence analysis of GP5 in the PRRSV JX03 and HN08 strains. The signal peptide, hypervariable region, and transmembrane domain are highlighted by black frames.

The nsp2 and GP5 proteins of PRRSV are among the most frequently mutated viral proteins, making them commonly used for phylogenetic analysis [43]. The amino acid sequences of nsp2 and GP5 from the JX03 and HN08 strains were aligned and analyzed against the reference sequences of PRRSV. The results revealed that the JX03 strain exhibits 30-aa deletions (1 + 29) in its nsp2 compared to the PRRSV-2 prototype strain VR-2332, which aligns with the deletion pattern observed in the HP-PRRSV reference strain JXA1. In contrast, the HN08 strain’s nsp2 contains 131-aa deletions (111 + 1 + 19), consistent with the deletion pattern of the NADC30 strain (Figure 5A). Moreover, the amino acid sequence alignment of the GP5 protein revealed that the JX03 strain was most homologous to the JXA1 strain, with a single mutation at position 195 (L195R). The HN08 strain showed high homology compared to the NADC34 PRRSV, harboring five mutations, including F25L, N32S, A98T, I161V, and V189I (Figure 5B). These findings agree with the results of the phylogenetic and homology analyses.

### 3.6. Analysis of Clinical Symptoms and Viral Loads in Piglets Infected with PRRSV

**Figure 6:**
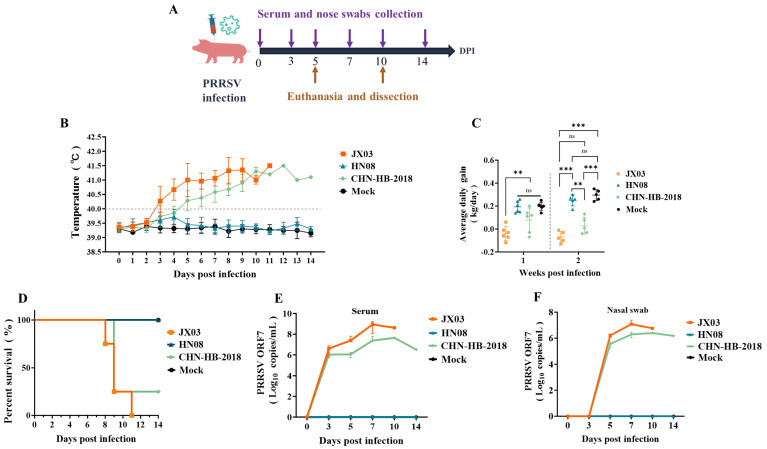
Clinical symptoms of different PRRSV strains in piglets. (**A**) Experimental procedure for the pathogenicity assessment of PRRSV in piglets. (**B**) Rectal temperature; (**C**) average weight gain; (**D**) percentage of surviving piglets in each group; (**E**) viral loads in serum; (**F**) viral loads in nose swabs. Error bars indicate the standard error based on at least three independent experimental replicates. Values in are shown as mean ± SD. ** *p* < 0.01; *** *p* < 0.001; ns: no significance.

We evaluated the pathogenicity differences between the JX03 strain (HP-PRRSV, lineage 8) and the HN08 strain (NADC34-like PRRSV, sublineage 1.5) in piglets. The NADC30-like PRRSV (sublineage 1.8) CHN-HB-2018 strain, which was previously isolated by our lab [44], served as a control, alongside a negative control group that did not receive a virus inoculation (mock). The experimental design is illustrated in Figure 6A.

Rectal temperature monitoring revealed that the JX03 strain infection caused a fever (over 40 °C) starting at 3 dpi and peaking at 42.3 °C, while the CHN-HB-2018 strain infection caused a fever at 5 dpi, peaking at 41.5 °C. In contrast, the HN08 strain group and the mock group maintained normal temperatures throughout the experimental period (Figure 6B). Body weight measurements and average daily gain (ADG) calculations were performed weekly after PRRSV infection. During the first week post-infection (wpi), the piglets infected with the JX03 strain showed a significantly lower ADG than the other three groups. And no significant differences in ADG were observed between the HN08 strain, CHN-HB-2018 strain, and mock groups. In the second wpi, ADG in both the JX03 and CHN-HB-2018 strain groups was significantly lower than those in the HN08 strain and mock groups. However, there were no significant differences between the JX03 and CHN-HB-2018 groups or between the HN08 strain and mock groups (Figure 6C). Additionally, piglets infected with the JX03 and CHN-HB-2018 strains had a survival rate of 0% and 25%, respectively. No mortality was observed in the HN08 strain or mock groups (Figure 6D).

Viral loads in serum and nasal swabs were determined using RT-qPCR for PRRSV ORF7. Viral ORF7 was detectable in both the JX03 and CHN-HB-2018 strain groups starting at 3 dpi in serum and at 5 dpi in nasal swabs, with levels gradually increasing over time and peaking at 7 and 10 dpi, respectively (Figure 6E,F). Notably, the JX03 strain group exhibited higher viral loads compared to the CHN-HB-2018 strain group. In contrast, viral RNA was not detected in piglets infected with the HN08 strain or in mock groups. To investigate differential tissue tropism among PRRSV isolates, viral loads in various tissues were compared at 5 and 10 dpi. The results showed that viral loads at various time points in the HN08 strain group were significantly lower than those observed in the JX03 and CHN-HB-2018 strain groups, consistent with the viral load patterns found in serum and nasal swabs.

**Figure 7:**
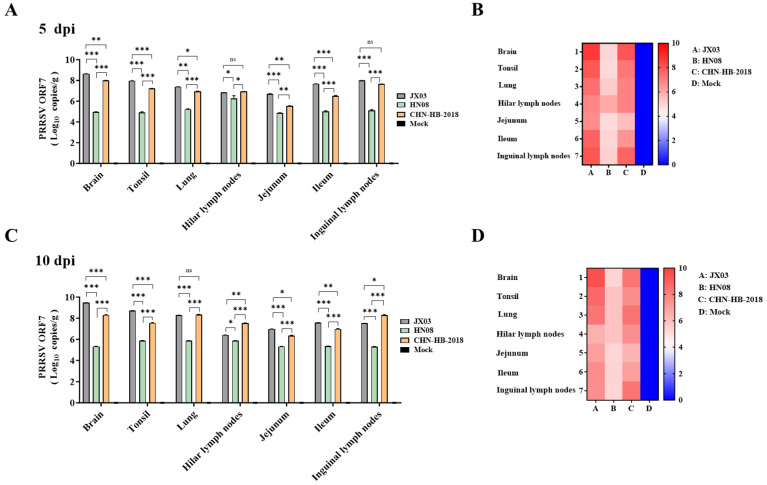
Analysis of viral loads in different tissues. (**A**,**B**) Viral loads in various tissues of piglets on 5 dpi. (**C**,**D**) Viral loads in various tissues of piglets on 10 dpi. Error bars indicate the standard error based on at least three independent experimental replicates. Values in are shown as mean ± SD. * *p* < 0.05; ** *p* < 0.01; *** *p* < 0.001; ns: no significance.

Specifically, in the JX03 and HN08 strain groups, the viral loads in the brain tissues and hilar lymph nodes were higher, respectively, at both 5 and 10 dpi (Figure 7A,C). As for the CHN-HB-2018 group, the viral load was highest in brain tissue at 5 dpi (Figure 7A,B) but the highest in lung tissues at 10 dpi (Figure 7C). We also constructed heatmaps based on the RT-qPCR results shown in Figure 7A,C, which more distinctly illustrate the differences in tissue tropism among piglets infected with different PRRSV strains (Figure 7B,D).

### 3.7. Pathological Analysis of Lungs and Hilar Lymph Nodes in Piglets

**Figure 8:**
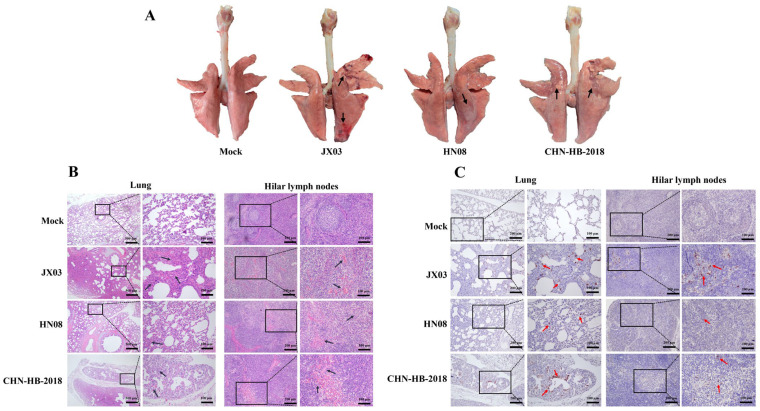
Pathological changes in PRRSV-infected piglets on 10 dpi. (**A**) Gross lesions in the lungs. (**B**) Pathological changes in lung and hilar lymph nodes of piglets (HE staining). (**C**) IHC analysis of lungs and hilar lymph nodes. Black arrow: tissue damage of lungs or erythrocyte or neutrophilic infiltration; red arrow: positive signals of PRRSV.

One piglet from each group was euthanized and necropsied at 5 and 10 dpi. The necropsy at 5 dpi indicated that piglets infected with the JX03 and CHN-HB-2018 strains exhibited focal parenchymal lesions in the lungs. In contrast, the piglets in the HN08 strain displayed interstitial damage, while obvious pathological lesions were observed in the mock group (Figure S1A). The lung lesions in the JX03 and CHN-HB-2018 groups worsened, characterized by widened interstitial lung tissue, pronounced consolidation, and accompanying hemorrhage at 10 dpi. In contrast, the piglets in the HN08 group presented with pulmonary edema. No obvious pathological lesions were observed in the mock group (Figure 8A).

Histopathological examinations using HE staining were conducted on the lungs and hilar lymph nodes. All the PRRSV infection groups exhibited bronchial hyperplasia and thickening of the alveolar walls in the lungs at 5 dpi, while the hilar lymph nodes showed no obvious lesions (Figure S1B). However, the results at 10 dpi revealed that extensive infiltration of the inflammatory cells and pulmonary hemorrhage were severe pathological changes that could be observed in the lung tissues of both the JX03 and CHN-HB-2018 groups (Figure 8B). In addition to these common pathological changes, the alveolar structure was nearly absent in the JX03 infection group, whereas in the CHN-HB-2018 infection group, although many alveoli had collapsed, a few relatively intact alveolar structures remained (Figure 8B). In contrast, most alveolar structures remained intact in the HN08 infection group, showing only mild thickening of the alveolar walls and some bronchial hyperplasia notes (Figure 8B). As for the hilar lymph nodes, the JX03 and CHN-HB-2018 strains caused lymphoid follicle atrophy and the infiltration of red blood cells in the lymphoid medulla and sinuses. Additionally, the hilar lymph nodes of piglets in the HN08 infection group exhibited only sparse lymphocytes in certain areas. No obvious pathological lesions were observed in the lungs and hilar lymph nodes of the mock group (Figure 8B). Collectively, the JX03 and CHN-HB-2018 strains exhibited severe lung and hilar lymph node lesions, whereas the HN08 strain showed mild damage.

IHC analysis was conducted to assess PRRSV antigen levels in the lung tissues and hilar lymph nodes of piglets. PRRSV-positive signals were detected in the lung tissues of piglets infected with both the JX03 and CHN-HB-2018 strains at 5 and 10 dpi, with the positive signal numbers increasing over time (Figure S2). In contrast, a limited number of positive signals were observed in the lung tissues of piglets infected with the HN08 strain at 10 dpi, while no positive signals were observed at 5 dpi (Figure 8C, Figures S1C and S2). These results indicated that the PRRSV antigen levels in piglets infected with the JX03 strain were higher than in the other groups, which was consistent with the findings from RT-qPCR. Furthermore, the PRRSV-positive signals were also detected in the hilar lymph nodes of piglets infected with all three PRRSV infection groups at 5 and 10 dpi, with higher signal numbers observed in the JX03 and CHN-HB-2018 strain groups compared to the HN08 strain group (Figure 8C and Figure S2).

## 4. Discussion

Since the first report of PRRSV in China in 1996, multiple strains have emerged, including classical PRRSV, HP-PRRSV, NADC30-like PRRSV, and NADC34-like PRRSV strains [18,34,45]. Gao et al. analyzed PRRSV strains circulating in China from 1996 to 2016 and found that HP-PRRSV accounted for 85.5% (2079/2430) of cases, while NADC30-like PRRSV represented only 5.2% (126/2430) [46]. However, a more recent epidemiological surveillance survey revealed that NADC30-like PRRSV had a positive rate of 74.18% in clinical samples, followed by an NADC34-like PRRSV detection rate of 11.98% and HP-PRRSV at just 4.04% [34]. These findings suggested that NADC30/34-like PRRSV is displacing HP-PRRSV as the predominant strain in China. Our study indicated that NADC30/34-like PRRSV strains are currently the primary circulating strains in China, accounting for 60.1%, which is in accordance with the findings of Xu et al. [34].

Previous studies have demonstrated that PAMs serve as the primary target cells for PRRSV infection and PAMs can successfully isolate field strains [47,48]. Notably, an increasing number of NADC30/34-like PRRSV strains demonstrate reduced or negligible infectivity in MARC-145 cells, whereas researchers have successfully isolated these strains using PAMs [49]. Consistent with these findings, PRRSV strains isolated by our lab between 2023 and 2025 exhibited similarly poor adaptability to MARC-145 cells (unpublished data). However, PAMs are primary cells that present several challenges, including high procurement costs, complex isolation procedures, and heightened susceptibility to contamination. Consequently, many research teams have employed alternative cell lines such as MARC-145, MA-104, and CL2621 for in vitro PRRSV isolation and propagation [50,51]. Therefore, it is significant to elucidate the mechanisms underlying the differential cytopathogenicity of PRRSV strains. Recently, several researchers have successfully adapted PRRSV to MARC-145 cells by replacing structural proteins or introducing mutations at the key amino acid sites of GP2a [52,53]. These advancements are crucial for the successful isolation and identification of newly emerging field strains of PRRSV.

The frequent recombination of PRRSV is a key factor contributing to the increasingly complex epidemiological landscape of the virus [54]. As a newly evolved branch of lineage 1, NADC34-like PRRSV shares recombination traits with NADC30-like PRRSV. For example, the GD-H1 strain exhibited the same “111 + 1 + 19” aa deletion pattern in nsp2 as NADC30-like strains, while the phylogenetic analysis of its ORF5 gene classified it within NADC34-like sublineage 1.5 [55]. The PRRSV strain SDLY23-1742 was confirmed as a multiclade recombinant of NADC30-like, NADC34-like, and HP-PRRSV strains [24]. In our study, the NADC34-like HN08 strain exhibited similar recombination characteristics to the previously reported GD-H1 strain [55]. Nucleotide homology analysis of the HN08 strain revealed that the nsp2 gene shares the highest sequence identity with the NADC30 strain, while the ORF5 gene is more closely related to the NADC34 strain, so it is defined as an NADC34-like strain. Given the frequent recombination in the nsp2 region of NADC30/34-like PRRSV, incorporating both nsp2 and ORF5 genes as reference markers in clinical PRRSV strain detection could significantly enhance diagnostic accuracy.

Current research consistently demonstrates that infection with HP-PRRSV induces marked fever in piglets, often leading to high mortality, whereas NADC30/34-like PRRSV typically results in milder clinical manifestations [24,31,33,44,53]. Consistent with these findings, our study revealed that the JX03 strain causes high morbidity and mortality rates in piglets. In contrast, piglets infected with the CHN-HB-2018 or HN08 strains exhibited milder clinical symptoms and showed lower or no mortality. However, PRRSV exhibits significant pathogenicity heterogeneity among NADC34-like PRRSV strains [24,56,57]. For instance, the NADC34 strain, first isolated in the U.S. in 2014, demonstrated high pathogenicity in piglets, characterized by high fever among other clinical signs [29]. The FJGD01 strain, isolated in Fujian as a recombinant of NADC30-like, NADC34-like, and JXA1-like PRRSVs, induced high viremia, prolonged fever, severe weight loss, and extensive pulmonary lesions in piglets [56]. In contrast, several other NADC34-like isolates displayed attenuated pathogenicity, causing only persistent fever, moderate respiratory distress, and mild interstitial pneumonia [58]. In addition to differences in pathogenicity, various PRRSV strains may exhibit distinct tissue tropism. Notably, PRRSV has been detected in the intestinal tracts of pigs, suggesting its potential enteric tropism [59]. Furthermore, Zhao et al. demonstrated that PRRSV infection induces intestinal barrier dysfunction in neonatal piglets and confirmed active viral replication in small intestinal tissues [60]. Consistent with these findings, our study revealed that PRRSV strains from different lineages could be detected in the intestinal tract of piglets, although differences in viral loads were observed.

## 5. Conclusions

In this study, we analyzed the epidemiological situation of PRRSV in China and found that the prevalent strains are primarily NADC30/34-like PRRSV, followed by HP-PRRSV. We successfully isolated and characterized an HP-PRRSV strain (JX03) and a recombinant NADC34-like PRRSV strain (HN08). Phylogenetic, amino acid sequence, and recombination analyses demonstrated that the JX03 and HN08 strains cluster within lineage 8 and sublineage 1.5, respectively. Animal experiments indicated that JX03 exhibited the highest pathogenicity, followed by the CHN-HB-2018 strain, while the HN08 strain exhibited the lowest pathogenicity. These findings are expected to aid in PRRSV prevention and control and support vaccine development efforts.

## Figures and Tables

**Table 1:** The RT-PCR detection results for PRRSV in China (2022–2023).

Clinical Samples	Positive Numbers			Positive Samples	Positive Rate	Percentage ^a^		
	HP- PRRSV	NADC30/ 34-like	Classical			HP- PRRSV	NADC30/ 34-like	Classical
1044	118	187	14	311	29.8%	37.94%	60.13%	4.50%

^a^: percentage of different PRRSV strains in positive samples.

**Table 2:** Homology analysis of the JX03 strain and reference PRRSV strains.

JX03	CH-1a		CH-1R		JXA1		NADC30		NADC34		GM2		QYYZ	
	nt	aa	nt	aa	nt	aa	nt	aa	nt	aa	nt	aa	nt	aa
Complete Genome	94.3		94.1		99.0		81.8		81.7		86.3		86.4	
5′UTR	96.8		95.3		99.5		92.1		90.0		94.7		94.7	
nsp1	94.3	92.4	94.2	92.4	99	98.7	84.8	85.4	84.4	87.2	88	89.1	88.5	89.1
nsp2	86.5	78.4	86.7	78.6	98.9	97.3	63.7	46.3	66.3	50.3	77.3	62.8	77.5	62.9
nsp3	94.8	98.0	94.8	97.8	99.0	98.9	83.3	90.6	82.8	90.8	81.8	88.1	82.0	88.1
nsp4	94.9	96.1	95.1	96.6	98.4	97.5	84.2	92.6	85.5	93.1	84.8	92.2	84.6	91.7
nsp5	94.5	94.1	93.9	93.5	98.9	98.8	90.0	92.9	84.3	88.8	82.7	90.0	82.4	90.0
nsp6	94.5	94.1	93.9	93.5	98.8	98.8	90.0	92.9	84.3	88.8	82.7	90.0	82.4	90.0
nsp7	94.5	94.1	93.9	93.5	98.8	98.8	90.0	92.9	84.3	88.8	82.7	90.0	82.4	90.0
nsp8	97.1	100.0	97.1	100.0	97.8	100.0	89.1	95.7	88.4	93.5	92.8	100.0	92.0	100.0
nsp9	96.4	98.6	96.3	98.3	98.9	99.1	87.2	97.2	87.4	96.9	98.9	99.1	91.3	96.9
nsp10	95.5	97.7	95.4	97.7	99.5	99.1	86.0	95.5	85.8	95.2	90.3	96.4	90.6	94.3
nsp11	94.9	96.9	95.2	97.8	99.0	99.1	91.2	96.4	87.2	95.5	89.0	94.6	88.8	93.8
nsp12	95.9	97.4	95.9	97.4	98.3	99.5	89.4	96.1	82.7	91.6	87.3	96.8	86.6	96.1
ORF2	96.4	96.1	95.7	95.7	99.2	98.8	86.5	86.4	87.4	86.0	89.9	88.7	89.9	89.1
ORF3	95.3	92.5	95.0	92.9	99.0	98.0	83.7	82.0	84.2	83.5	90.6	89.8	90.8	89.4
ORF4	96.5	96.5	96.3	96.3	97.6	97.6	87.5	87.5	86.8	86.8	95.0	95.0	94.8	94.8
ORF5	94.9	93.0	94.0	91.5	99.3	99.5	85.7	86.1	87.1	87.1	83.3	82.1	83.6	82.6
ORF6	97.3	97.1	97.0	96.6	99.8	99.4	89.1	93.7	89.3	94.3	91.6	96.6	91.0	97.1
ORF7	95.7	94.4	95.7	95.2	99.5	98.4	90.6	90.3	89.2	90.3	87.9	88.7	89.2	91.1
3′UTR	96.1		97.2		99.4		90.4		89.9		88.8		89.9	

**Table 3:** Homology analysis of the HN08 strain and reference PRRSV strains.

HN08	CH-1a		CH-1R		JXA1		NADC30		NADC34		GM2		QYYZ	
	nt	aa	nt	aa	nt	aa	nt	aa	nt	aa	nt	aa	nt	aa
Complete Genome	82.7		82.8		82.5		90.0		85.3		79.6		79.8	
5′UTR	90.8		89.2		90.3		96.4		89.7		88.2		88.2	
nsp1	81.2	83.6	81.3	83.6	80.5	83.9	91.0	91.1	83.2	84.9	79.2	82.8	79.7	82.6
nsp2	60.6	42.2	60.8	42.4	61.0	42.7	90.7	80.6	74.5	55.5	59.6	40.0	59.6	40.3
nsp3	84.4	92.6	84.5	92.4	84.3	91.7	89.8	94.8	84.2	90.8	79.7	87.4	79.6	87.4
nsp4	92.3	95.1	92.5	95.6	94.3	96.6	84.2	92.6	84.0	94.6	84.0	92.2	83.8	91.7
nsp5	90.0	92.4	89.8	91.8	92.7	93.5	87.3	92.4	81.6	88.8	81.2	90.0	80.8	90.6
nsp6	91.7	100.0	91.7	100.0	93.8	100.0	87.5	93.8	81.2	87.5	91.7	100.0	91.7	100.0
nsp7	94.5	95.0	94.1	94.6	97.4	98.1	81.5	83.8	81.2	85.3	90.3	91.1	92.7	94.2
nsp8	97.8	100.0	97.8	100.0	97.1	100.0	89.9	95.7	89.1	93.5	93.5	100.0	92.8	100.0
nsp9	91.8	98.0	91.7	97.7	92.7	98.1	90.8	97.7	87.5	96.9	89.3	97.0	88.5	96.6
nsp10	84.2	93.2	84.2	93.2	83.8	93.4	94.1	97.5	89.8	97.0	84.8	94.8	83.6	93.6
nsp11	89.9	95.5	90.2	96.4	87.8	96.0	93.9	97.3	85.6	94.6	85.7	93.8	85.6	92.9
nsp12	88.6	94.8	88.8	94.8	88.8	95.5	93.5	96.8	85.5	90.3	86.8	94.8	86.4	94.2
ORF2	85.1	82.9	85.1	82.5	84.8	81.3	83.7	81.7	94.8	92.2	84.4	81.3	84.6	81.7
ORF3	83.8	80.4	84.1	80.8	83.4	81.2	84.6	83.1	92.9	92.9	83.4	81.6	83.1	82.4
ORF4	86.4	88.3	86.4	87.7	85.5	87.2	94.8	95.5	94.8	96.6	85.5	86.0	85.7	86.6
ORF5	86.4	88.1	86.1	86.6	86.2	89.6	87.2	91.0	95.9	97.0	83.6	84.1	83.6	83.6
ORF6	86.9	92.6	86.9	92.0	87.2	93.1	94.7	96.0	94.3	95.4	89.7	93.1	88.8	93.1
ORF7	90.3	91.9	90.3	91.9	90.3	90.3	95.4	95.2	93.8	92.7	84.9	86.3	86.6	89.5
3′UTR	89.3		88.8		90.4		97.8		95.5		88.8		89.9	

## Data Availability

The data presented in this study are available upon request from the corresponding author.

## Supplementary Materials

The following supporting information can be downloaded at: https://www.mdpi.com/article/10.3390/vetsci12060530/s1, Figure S1: Pathological changes in PRRSV-infected piglets on 5 dpi.; Figure S2: Quantitative comparison of the PRRSV-positive signals in the lungs and hilar lymph nodes of piglets across different groups; Table S1: Primer sequences used for the amplification of the whole-genome of the JX03 and HN08 strains;; Table S2: The reference strains used for PRRSV sequence alignment.
