# Supplementary material for: Etiological Detection, Isolation, and Pathogenicity of Porcine Reproductive and Respiratory Syndrome Virus in China
Source: Vet Sci. 2025 May 29;12(6):530. doi: 10.3390/vetsci12060530 (PMC12197495; doi:10.3390/vetsci12060530)
Supplement: Supplementary file 1 [file vetsci-12-00530-s001.zip › vetsci-3649681-supplementary.pdf]

Supplementary Figure S1

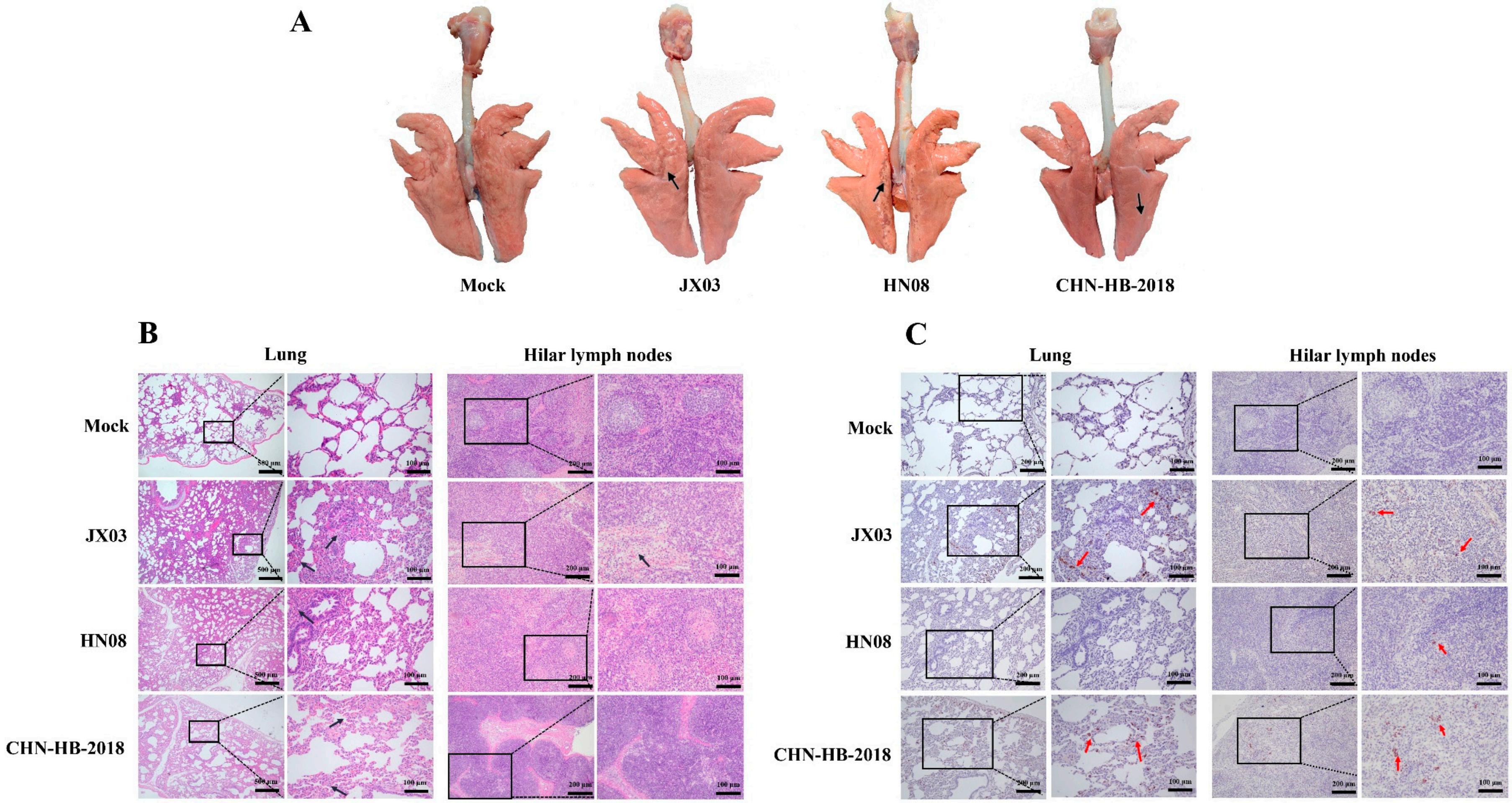

Supplementary Figure S1. Pathological changes of PRRSV-infected piglets on 10 dpi. **(A)** Gross lesions in the lungs. **(B)** Pathological changes in lung and hilar lymph nodes of piglets (HE staining). **(C)** IHC analysis of lungs and hilar lymph nodes. Black arrow: tissue damage of lungs, erythrocyte or neutrophilic infiltration, red arrow: the positive signals of PRRSV.

Supplementary Figure S2

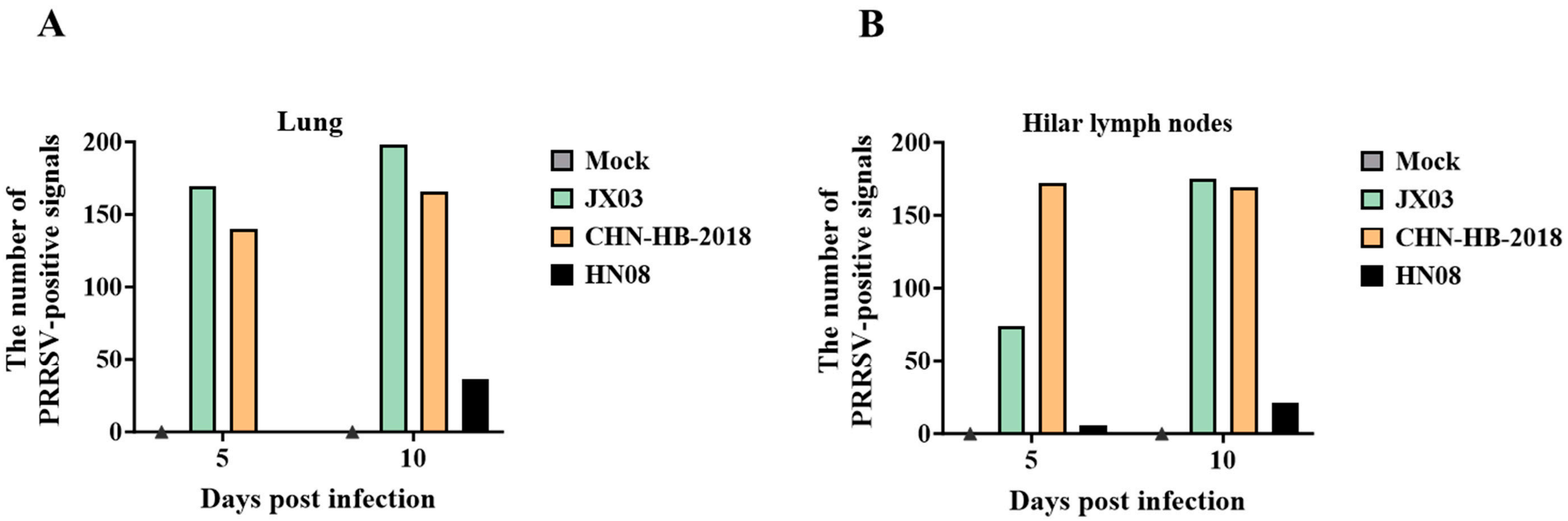

Supplementary Figure S2. Quantitative comparison of the PRRSV-positive signals in the lungs and hilar lymph nodes of piglets across different groups. **(A)** PRRSV-positive signals in the lungs. **(B)** PRRSV-positive signals in the hilar lymph nodes. Black triangle: no PRRSV-positive signals in the mock group.

Supplementary Table S1 Primer sequences used for the amplification of the whole-genome of the JX03 and HN08 strains.

NO.	Primers	Sequences (5′-3′)	Location	Length (bq)
1	JX03-1F	ATGACGTATAGGTGTTGGCTCTAT	1-24	1287
2	JX03-1R	CAGTGGTGACGTATTGGGCTCAA	1265-1287	
3	JX03-2F	GAGGCTGCAAGTTAATGGTCTT	1113-1134	1282
4	JX03-2R	TAACCTCTCACGGTGATGAACCT	2372-2394	
5	JX03-3F	AACCTCACGTCAACTCATGCT	2249-2269	1294
6	JX03-3R	GTGTCTCCTATTTTCCCGAGGATG	3519-3542	
7	JX03-4F	TCTCCCAAAGATGATTCTCGAGACA	3384-3408	1207
8	JX03-4R	CTGTAATACCCGCAAGCACTTTAC	4567-4590	
9	JX03-5F	TTGAGCAACCTTCTGAAAAACCCATC	4466-4491	

10	JX03-5R	ATGCCTCCTCCTTGTTTGTTTG	5933-5954	1489
11	JX03-6F	GTTGAACCCGGTGTTATTGGGAAT	5821-5844	1564
12	JX03-6R	CAATGGTGATATGCCCACACTGAA	7361-7384	
13	JX03-7F	AGTACCAGAAATTTTGGGACAAGAATT	7238-7264	1619
14	JX03-7R	AGGCGAGTTCATAAAGAAGATTGGC	8832-8856	
15	JX03-8F	AAAAAAGCGTTCAACTCGCCCA	8694-8715	1500
16	JX03-8R	GGAGTTCTTGCCAGGACACCAACC	10170-10193	
17	JX03-9F	CATGCTCGACATGATTAGGGCTTTG	10055-10079	1539
18	JX03-9R	TACACAGTAGAATTAACAGGAACTCG	11568-11593	
19	JX03-10F	CTTTAAGGAGGTTCGACTGATGGTATG	11462-11488	1537
20	JX03-10R	ATTCCCTATCCCAAATATCTCGGGAT	12973-12998	
21	JX03-11F	CTAGGGTTCATGGTTCCGCCT	12873-12893	

22	JX03-11R	TCAAAAGGTGCAGAAGCCCTAGCA	14406-14429	1557
23	JX03-12F	CGACTTCTGCAATGATAGCACAGC	14302-14325	1032
24	JX03-12R	TTTTTTTTTTTTTAATTACGGCCGC	15309-15333	
25	HN08-1F	TATGACGTATAGGTGTTGGCTC	1-22	1482
26	HN08-1R	GGGAGTAGTACTTGAGGAGCT	1462-1482	
27	HN08-2F	GTTTGAGGACCTCCTCAGAATA	1241-1262	1687
28	HN08-2R	GTATTTCTCCTTTACCTCTTGGAG	2904-2927	
29	HN08-3F	CGAGCTCCTCGATTTGTCT	2651-2669	1336
30	HN08-3R	CTTTTTACACCTACCTTGCGAA	3965-3986	
31	HN08-4F	GTTCTTGGCAGGTTACTGGG	3867-3886	1332
32	HN08-4R	ATGGTGCGACCAGTCAACG	5170-5188	
33	HN08-5F	AATGTTGCCGGTCTTGTCAC	5052-5071	

34	HN08-5R	CTTCAAGTCGAAGATGCTGC	6679-6698	1647
35	HN08-6F	TCCTGGCCAAACTTGAAGC	6589-6607	1040
36	HN08-6R	CAAAATCCCAAAGCGTGCCA	7871-7895	
37	HN08-7F	TGGCCAGTGAGGTCGAGC	7425-7442	1512
38	HN08-7R	CAGTGATAGCTGTCTTCTTTGGG	8914-8936	
39	HN08-8F	CAAGTTTGAGGACATGCTTAAG	8776-8797	1781
40	HN08-8R	TCGACCCTTCCAGATCTG	10539-10556	
41	HN08-9F	GATGGGCAACTGATTGTGCT	9614-9637	1745
42	HN08-9R	TACAAGTCTCGGCTTCAATGG	12139-12159	
43	HN08-10F	TGATGAAATGGTGTCGCGAC	12002-12021	1105
44	HN08-10R	GTCACGTTGGCTGTGACAG	13088-13106	
45	HN08-11F	GCGTTTCGCAAAATCCCTC	13033-13051	

46	HN08-11R	GTGATGAATCTCCAGGTTTCCAT	14248-14270	1238
47	HN08-12F	CACTTTTGGGTATATGACATTTGT	14148-14171	873
48	HN08-12R	AATTTCGGCCGCATGGTT	15003-15020	

Supplementary Table S2 The reference strains used for PRRSV sequence alignment.

NO.	Strains	Reported	GenBank No.	NO.	Strains	Reported	GenBank No.
1	VR2332	USA 1992	U87392	36	10-FUJ-1	China 2010	JQ663546
2	Resp PRRS MLV	USA 1994	AF066183	37	10-FUJ-2	China 2010	JQ663547
3	SDSU73	USA 1996	JN654458	38	SX-1	China 2009	GQ857656
4	JA142	USA 1997	AY424271	39	NT0801	China 2008	HQ315836
5	NADC30	USA 2008	JN654459	40	TJbd14-1	China 2014	KP742986
6	NADC31	USA	JN660150	41	YD	China	JF748717

		2008				2009	
7	CH-1a	China	AY032626	42	JL-04/12	China	JX177644
		1996				2012	
8	CH-1R	China	EU807840	43	GX1001	China	JQ955657
		2008				2010	
9	BJ-4	China	AF331831	44	HB-1(sh)/2002	China	AY150312
		1996				2001	
10	HN1	China	AY457635	45	HB-1/3.9c	China	HQ233605
		2003				2002	
11	JXA1	China	EF112445	46	MN184A	USA	DQ176019
		2006				2001	
12	JXA1-p80	China	FJ548853	47	HeNws16	China	MF474322
		2008				2016	

13	JXA1-P120	China 2009	KC422727	48	HENAN-HEB	China 2013	KF416334
14	JXA1-P170	China 2009	JQ804986	49	FJXS15	China 2015	KX758250
15	HuN4	China 2006	EF635006	50	CH/SCLS-2/2020	China 2020	OL771207
16	TJ	China 2006	EU860248	51	CH/SCMS-4/2020	China 2020	OL771208
17	HEB1	China 2006	EF112447	52	FJ0908	China 2018	MK202794
18	R98	China 2006	DQ355796	53	IA/2014/ISU-8	USA 2014	MF326995
19	GD	China	EU825724	54	IA/2014/NADC34	USA	MF326985

		2007				2014	
20	WUH3	China 2008	HM853673	55	IA/2015/ISU-10	USA 2015	MF326997
21	YN-2011	China 2011	JX857698	56	IA/2015/NADC35	USA 2015	MF326986
22	HZ-31	China 2012	KC445138	57	IA/2015/NADC36	USA 2015	MF326987
23	SD16	China 2012	JX087437	58	ISU17	USA 2014	KT257967
24	FJFS	China 2012	KP998476	59	HLJTZJ2260	China 2020	OL546288
25	NT1	China 2012	KP179402	60	JLWK412	China 2020	OL546281

26	YN9	China 2008	GU232738	61	HLJWK475	China 2020	OL546283
27	GM2	China 2011	JN662424	62	SDTZJ2039	China 2020	OL546251
28	QYYZ	China 2011	JQ308798	63	JSTZJ2028	China 2020	OL546250.
29	JL580	China 2015	KR706343	64	Ingelvac ATP	USA 1999	DQ988080
30	CHsx1401	China 2014	KP861625	65	QY2010	China 2010	JQ743666
31	10-10HEB-3	China 2010	JQ663553	66	HNyc15	China 2015	KT945018
32	Shanxi-6	China	KJ855518	67	CH2002	China	EU880438

		2010				2009	
33	HN-09	China 2009	JX174280	68	HH08	China 2011	JX679179
34	09HEB	China 2009	JF268679	69	GX1003	China 2010	JX912249
35	09HEN1	China 2009	JF268684	70	HENZK-2	China 2014	KT424218
